# Case of Instrumental Labor, with Puerperal Convulsions

**Published:** 1867-07

**Authors:** V. H. Taliaferro

**Affiliations:** Columbus, Ga.


ATLANTA

Medical and Surgical Journal.

NEW SERIES.

Vol. VIII. JULY, 1867. No. 5.

### ORIGINAL COMMUNICATIONS.

### ARTICLE I.

*Case of Instrumental Labor, with Puerperal Convulsions. Beneficial effects of the Hypodermic Syringe.* By V. H. Taliaferro, M. D., Columbus, Ga.

Some months since, I was called to see a young woman in labor with her first child. Upon my arrival, about 6 o'clock A. M., I found her suffering from the most violent and protracted convulsions. I learned from the old midwife in attendance, that the convulsions had existed almost simultaneously with the commencement of labor, some six or eight hours previous, up to the present time, recuring with great rapidity and violence, and at no time in the short intervals of the convulsions, giving the least evidence of conscienceness.

An examination per vaginum, revealed a vertex presentation—the head well impacted in the superior strait.

From the alarming condition of this woman, together with the length of time her convulsions had existed, I could certainly give but little encouragement as to her recovery. I immediately commenced the administration of chloroform, with which to control *completely*, the convulsions. I had in the mean time sent for my friend, Dr. M. J. Moses, with the request that he bring with him some obstetrical forceps; feeling convinced that the only hope for the mother's safety lay in a speedy delivery. Upon the Doctor's arrival, the convulsions still not having been controlled, he suggested bleeding from the arm, to which I readily assented. She was therefore at once bled, and with the most satisfactory result; the convulsions afterwards being easily controlled by the moderate use of chloroform. The forceps were now applied, and after continued and persevering efforts for more than an hour, but little progress had been made towards delivery. The operation of craniotomy was now determined upon, as giving the only remaining chance for the mother. Instruments for the purpose were accordingly sent for, they being close at hand, but fortunately before their arrival, the head was moved perceptibly from its firmly impacted position. Being now more encouraged that delivery would soon be effected with the forceps and loath to perform the operation of craniotomy, our efforts with the forceps were continued for more than an hour longer, when an unusually large child was delivered "still born." Near three hours had been consumed from the time of the application of the forceps to the period of delivery. From the long continued pressure upon the child's head, but little hope could be entertained for its resuscitation. Dr. Moses finally succeeded by means of artificial respiration, in restoring life to the apparently dead child, and in a little while it was crying as lustily, and doing as well as infants born under more favorable circumstances.

The placenta was now delivered, and the uterus left well contracted.

Our patient at this time, was certainly in a most alarming condition from profound coma and great prostration.

We now administered gr. i sulphate morphia, by *hypodermic injection*, and the patient left until the following evening, when the same operation was repeated. On the following morning the patient was found in much the same con dition, save some little improvement in the pulse and respiration. The hypodermic injections were repeated as on the previous day—morning and evening. In the afternoon of the third day, the morphia having been suspended, the woman aroused, sat up in bed and spoke to her attendants. For some time she could not be made to believe that she had been delivered, and that the fine healthy looking infant was her own.

From this time no untoward symptom presented itself, and recovery to perfect strength and health was more rapid, even, than is usual with women who have undergone the most natural and easy labor.

I consider the recovery of this woman, mainly due to the *hypodermic* administration of the sulph. morphia, whereby a sufficient narcotism was induced immediately following delivery, and kept up until re-action had quietly and firmly established itself, avoiding thereby the violent re-action which would in all reasonable probability have ensued, and resulted in the most active inflammation, establishing itself, most likely, in the peritonium, the uterus, or, indeed both.

